# The efficacy of superior oblique posterior tenectomy in the treatment of A-pattern exotropia without ocular intorsion: A retrospective study

**DOI:** 10.1186/s12886-019-1298-4

**Published:** 2020-01-21

**Authors:** Yan Wei, Ling-Yan Dong, Pei-quan Zhao, Xiao-li Kang

**Affiliations:** Department of Ophthalmology, Xinhua Hospital, School of Medicine, Shanghai Jiao Tong University, Shanghai, 200092 People’s Republic of China

**Keywords:** Superior oblique posterior tenectomy, A-pattern, Strabismus, Intorsion

## Abstract

**Background:**

Superior oblique weakening is a common method to treat A-pattern strabismus. This study aims to evaluate the surgical results of the bilateral superior oblique posterior tenectomy procedure to treat A-pattern strabismus patients who had bilateral superior oblique overaction without objective ocular intorsion.

**Methods:**

The records of 18 consecutive patients who underwent surgery of superior oblique posterior tenectomy close to its insertion with superior oblique overaction (SOOA)-associated A-pattern strabismus between September 1, 2015 and August 31, 2018 were retrospectively reviewed. Ocular alignment, objective torsion, A-pattern and ocular motility were assessed. Ocular alignment was measured in the primary position, 25° upgaze, and 25° downgaze using the prism bar cover test, and torsion was measured using fundus photographs.

**Results:**

A total of 18 patients (mean age: 15 years; 6 female, 12 male) underwent bilateral superior oblique posterior tenectomy and simultaneous horizontal rectus muscle surgery were included. The mean preoperative A-pattern deviation was 15 PD and the mean postoperative A-pattern deviation was 2.25 PD with a mean reduction of 12.75 PD. The mean preoperative superior oblique overaction was 2.28 and the mean postoperative superior oblique overaction was 0.43 with a mean reduction of 1.85. There was no significant correlation between the ocular torsional, vertical alignment change and the superior oblique posterior tenectomy procedure.

**Conclusions:**

Superior oblique posterior tenectomy surgery selectively improved the A-pattern and superior oblique overaction but not affect the primary position vertical deviation, as well as the ocular torsion. It is an effective procedure to treat the mild to moderate superior oblique overaction associated A pattern strabismus without ocular intorsion.

## Background

A-pattern strabismus is usually accompanied with superior oblique overaction, and superior oblique weakening is helpful to solve this situation. Superior oblique posterior tenectomy (SOPT) was proposed first in 1976 to correct A-pattern strabismus [1]. SOPT aims to weaken the abduction effect in downgaze through partial cutting the posterior fibers of superior oblique, and induce clinically insignificant changes in torsion by leaving the anterior fibers intact. However, it has been reported that the magnitude of A-pattern correction obtained by SOPT is less than that obtained by complete superior oblique tenotomy or tenectomy [2, 3], and few studies have evaluated concretely the A pattern, superior oblique function and torsional changes after SOPT [4, 5]. The purpose of this study was to evaluate the effect of SOPT on A-pattern extropia without ocular intorsion and its association with the primary position horizontal and vertical deviation to provide further information for the preoperative planning surgical technique of these patients.

## Methods

The clinical records of consecutive patients with A-pattern strabismus associated with bilateral superior oblique overaction from September 1, 2015 and August 31, 2018 were retrospectively reviewed, to identify patients who had undergone bilateral superior oblique posterior 3/4 tenectomy for A-pattern deviation without ocular objective intorsion during the study period, and had at least 3 months of postoperative follow-up.

The data collected included age, sex, preoperative and postoperative prism cover test at 33 cm and 6 m, A-pattern, ocular movement, objective torsion and history of previous strabismus surgery. An A pattern was defined as >10PD difference between up- and downgaze at 6 m by use of the alternate prism and cover test. A dilated fundus evaluation and fundus color photography were performed to evaluate objective torsion before and after surgery. To evaluate the extent of torsion, a line is drawn from the center of the disc to the center of the fovea. The angle formed between this line and the line passing through the center of the disc is the extent of the torsion and measured by coreldraw 11.0 (Corel Corporation). The average value of the three measurements is taken. SO overaction was measured on a 9-cardinal system, from − 4 to + 4. Exaggerated traction test was performed before and after SO posterior tenectomy. Patients who had previous strabismus surgery were excluded.

The surgical technique of SOPT involved rotating the globe inferiorly, the conjunctival incision was in the superior temporal bulbar conjunctiva at the lateral border of the superior rectus and 8 mm posterior to the superior limbus. The superior oblique tendon was isolated on a muscle hook to expose the whole posterior part of the tendon. The superior oblique posterior tenectomy extended from the insertion to the lateral border of superior rectus, and the anterior 1/4 of the superior oblique tendon was left intact.

Statistical analysis was carried out using SPSS software version 12.0 (SPSS Inc., Chicago, Illinois, USA). A paired t-test was used to determine the statistical significance between preoperative and 3 months postoperative results. *P* values less than 0.05 were considered statistically significant.

## Results

Eighteen patients (thirty-six eyes) were included in the study. There were 6 female and 12 male patients in age ranging from 3 to 41 years, with a mean age of 15.22 years. The mean of follow up time was 19.33 month (ranging, 15–25 months). All patients showed A-pattern exotropia and bilateral superior oblique overaction without intorsion, and underwent bilateral superior oblique posterior tenectomy with horizontal strabismus surgery. None of the patient had diplopia prior to surgery. The operation was aim to improve the cosmetic appearance and tried to reconstruct binocular single vision.

### A pattern

**Fig. 1:**
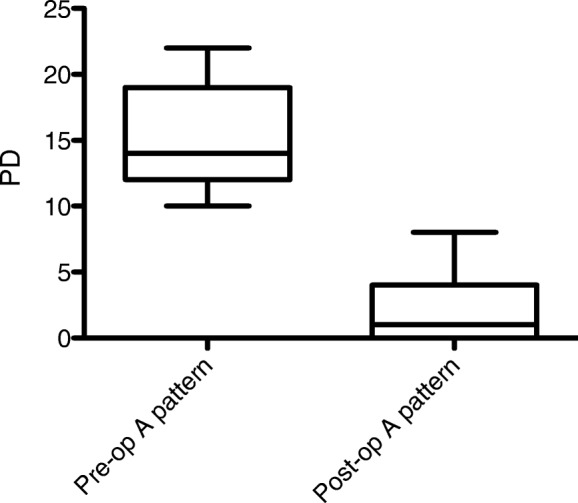
Box-and-whiskers plot demonstrating the median (range) values of the pre- and postoperative A-pattern size (*p* < 0.05). PD: prism diopters

Surgery was successful in collapsing the A pattern in all the patients. The mean A patterns were 15 ± 3.87PD preoperatively (range, 10PD-22PD) and 2.25 ± 2.73PD postoperatively (range, 0PD-8PD) (Fig. 1). There was 12.75PD (85%) reduction in the size of the A pattern postoperatively.

### Superior Oblique Overaction

**Fig. 2:**
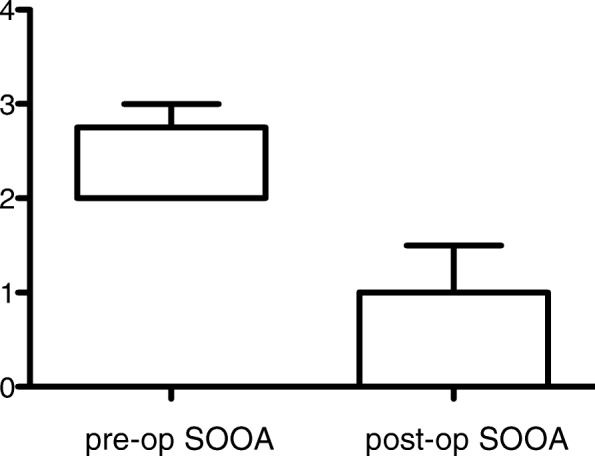
Preoperative and postoperative superior oblique overaction in patients who underwent, bilateral SO posterior tenectomy (mean and SD)

The mean superior oblique overaction preoperative was 2.28 ± 0.42 (range, 2 to 3), with a mean postoperative residual of 0.43 ± 0.49 (range, 0 to 1) (Fig. 2).

### Objective Torsional Deviation

**Fig. 3:**
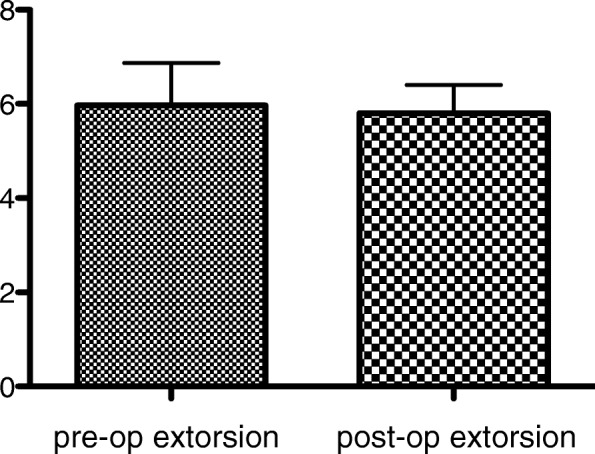
Preoperative and postoperative objective torsional deviation in patients who underwent, bilateral SO posterior tenectomy (mean and SD)

Based on the fundus examination, the mean preoperative extorsion was 5.97° ± 2.45° and postoperative extorsion was 5.8° ± 1.35° (Fig. 3). No patient who underwent SO posterior tenectomy showed significant fundus torsion change or complained of torsional diplopia after surgery.

### Primary position deviation

All the 18 patients showed primary position exotropia without vertical deviation at presentation, and had simultaneous surgery on their horizontal rectus muscles for exotropia. The mean exodeviations were 33.11 ± 13.21PD preoperatively (range, 8-50PD) and 4.11 ± 2.03PD postoperatively (range, 1-8PD). None of the patients showed postoperative primary position vertical deviation.

### Complications

In our series, no complications such as superior oblique palsy, postoperative V pattern, or induced extorsion were encountered.

## Discussion

Our study recruited 18 patients with an overall A-pattern of 15 PD for whom a correction of 12.75PD (85%) was achieved. The mean superior oblique overaction grade decreased from 2.28 before surgery to 0.43 after surgery.This was a statistically significant change. No induced vertical deviations were seen in those patients and no patient showed significant fundus torsion change or complained of torsional diplopia postoperatively. This study suggests that SOPT effectively collapses mild to moderate A-pattern deviation and superior oblique overaction, but not affect the primary position vertical deviation, as well as the ocular torsion.

Several techniques were able to weaken the superior oblique muscle, such as superior oblique tenotomy, tenectomy, posterior tenectomy, recession or tenotomy with a prosthetic spacer. The main complications associated with complete tenectomy or tenotomy are iatrogenic superior oblique palsy, conversion of an A -pattern to a V -pattern, or induced extorsion [6, 7]. SOPT selective weaken the abduction in downgaze by leaving the anterior fibers intact, and the risks of inducing superior oblique palsy, V-pattern or extorsion were not seen in our study. Therefore this procedure is an effective surgical approach for A pattern strabismus with superior oblique overaction but no intorsion.

## Conclusions

In our study, bilateral SOPT proved to be an effective treatment for mild to moderate SOOA associated A-pattern, without causing torsional deviation change and vertical deviation in primary position. Compared with complete superior oblique weakening techniques, SOPT spares the anterior fibers responsible for torsion, thus avoiding postoperative undesirable torsional complications. Further study may be necessary to answer those questions whether it can be helpful to treat patients with severe SOOA associated A-pattern deviation and ocular intorsion.

## Data Availability

The datasets used and/or analysed during the current study are available from the corresponding author on reasonable request.

## Abbreviations

SOOA: Superior oblique overaction
SOPT: Superior oblique posterior tenectomy
